# Student performance on the Test of Scientific Literacy Skills (TOSLS) does not change with assignment of a low-stakes grade

**DOI:** 10.1186/s13104-018-3545-9

**Published:** 2018-07-03

**Authors:** Verónica A. Segarra, Nicole M. Hughes, Kristin M. Ackerman, Michael H. Grider, Todd Lyda, Patrick A. Vigueira

**Affiliations:** Department of Biology, High Point University, One University Parkway, High Point, NC 27268 USA

**Keywords:** Test of Scientific Literacy Skills (TOSLS), Response-validated multiple-choice assessments, Low-stakes testing, No-stakes testing, Student effort

## Abstract

**Objective:**

Response-validated multiple-choice assessments are used in college courses to assess student learning gains. The ability of a test to accurately reflect student learning gains is highly dependent on the students’ effort. Within our institution, lackluster student effort is common on response-validated multiple-choice concept assessments that are not included as a portion of the semester grade but are used to inform curricular changes. Thus, we set out to determine whether increasing testing stakes by assigning a grade on student performance had an effect on student score and self-reported effort. The Test of Scientific Literacy Skills (TOSLS) is a response-validated multiple-choice assessment used to measure scientific literacy in undergraduates. We administered the TOSLS to students enrolled in a general education Biology course, both during the first 2 weeks (pretest) and the last 2 weeks (posttest) of the course.

**Results:**

Self-reported effort and TOSLS performance were significantly correlated in the ungraded cohort. This relationship did not exist in the graded sections. Our data indicate that assigning a low-stakes grade has no significant effect on mean student performance or self-reported effort on the TOSLS within our general education course.

## Introduction

In recent years, undergraduate institutions have sought to reform the curriculum of Biology courses according to recommendations from the American Association for the Advancement of Science Vision & Change (V&C) in Undergraduate Biology Education Initiative [1–3]. At our institution we have also incorporated V&C recommendations into general education Biology courses including inquiry-based laboratory modules that allow students to experience science as a process, opportunities to engage with the content through active and collaborative learning methodologies, and additional opportunities for science communication.

Our ultimate goal is for our general education Biology courses to help non-science majors develop scientific literacy, the ability to identify data generated from scientifically-sound studies, and the ability to organize, analyze, and draw conclusions from this data [4]. In 2012 Gormally et al. [4] developed the Test of Scientific Literacy Skills (TOSLS) as a resource for biology instructors to evaluate their students’ proficiencies in scientific literacy, including the identification and analysis of data that contributes to building scientific knowledge. We decided to use this tool to determine whether the curricular changes we were implementing were resulting in scientific literacy learning gains.

Alongside using the TOSLS to assess learning gains related to scientific literacy, we also wanted to consider the effects of student effort and motivation on TOSLS performance. The ability of an assessment to accurately reflect student learning gains is dependent on student effort [5–9]. A student bringing his/her best effort to a test should translate into a performance that accurately reflects his/her true abilities and knowledge about the particular subject [7]. Within our institution, lackluster student effort is common on no-stakes standardized assessments that are used by instructors to inform curricular changes but do not contribute to a course grade. While student performance on these assessments has no effect on their course grade, we use their assessment scores to determine whether students have had gains in areas like scientific literacy. For this reason, we set out to determine whether increasing the testing stakes by assigning a low-stakes grade on TOSLS student performance would have an effect on student score and self-reported effort on the TOSLS. We hypothesized that increasing the stakes for students taking the TOSLS assessment would lead to higher reported student effort and higher student TOSLS scores. In addition, we did not believe that an increase in effort would impact the degree to which the students’ TOSLS scores improved in the pretest/posttest analysis.

Before this study, the TOSLS was administered as a no-stakes assessment; student performance on the TOSLS was not factored into the course grade and was therefore considered by the students to be of little or no consequence. Under these circumstances, we often noticed what appeared to be a lack of effort from students while taking the TOSLS (e.g. highly abbreviated testing time, obvious distraction, verbal assertion of disinterest). Student effort is known to influence whether or not a test score reflects a student’s abilities and skills in a particular subject [10]. In fact, student effort can vary more widely in low-stakes testing [10]. For this reason, we sought to determine the effects of increased testing stakes on self-reported student effort and student TOSLS scores. We designed a controlled experiment in which the TOSLS was administered in a low-sakes testing environment; the TOSLS score was scaled to a mean of 90%, maximum score of 100%, and included as quiz grade (approximately 0.75–3% of the overall course grade). By using this grading policy, we attempted to establish a TOSLS grading policy that minimized testing anxiety in this inherently stressful situation to some students. We anticipated that students in graded sections may experience mild testing anxiety derived from their inability to specifically study or prepare for the TOSLS content.

## Main text

### Methods

#### Participants

Participants were volunteer students in a non-majors, general education Biology course at High Point University, NC, USA. Student participant demographics information was collected at the end of the post-assessments in an effort to minimize stereotype threat (Table 1). Students under the age of 18 were not allowed to participate in the study. A total of 14 course sections (164 students) participated in this study. Only data from consenting students that completed both the pretest and posttest were included in our analyses. These sections were taught by five instructors in the fall semester of 2015 and the spring semester of 2017. 7 of these sections (83 students) were not assigned a grade for their TOSLS performance, while 7 of these sections (81 students) were assigned a grade for their TOSLS performance. In order to minimize the impact of inter-instructor variability on our study, individual instructors were assigned nearly equal numbers of graded and non-graded sections.

**Table 1:** Demographic and class standing of participants in this study

	Gender		
	Female	Male	Other
No grade	68	15	0
Grade	57	24	0
Total	125	39	0

	Race					
	Asian	Black	Hispanic	Native American	White	Other
No grade	2	10	0	0	71	0
Grade	2	6	6	1	65	1
Total	4	16	6	1	136	1

	Class			
	Freshman	Sophomore	Junior	Senior
No grade	56	21	5	1
Grade	48	25	4	4
Total	104	46	9	5

Numbers correspond to number of individuals

#### Data collection

The TOSLS assessment was administered to participants in a pretest/posttest format in the first and last meetings of the laboratory course component of a non-majors, general education Biology course at High Point University, NC, USA. The TOSLS was administered using the *Qualtrics* (Provo, Utah, US) software system on the students’ personal laptop or tablet computers. The TOSLS contains 28 multiple choice questions with a time limit of 35 min. Following a brief introduction to the assessment instrument and our study, students simultaneously initiated the timed portion of the test and were given a verbal notification when 5 min remained in the testing period. Immediately following the completion of the TOSLS, all students were asked to complete a short survey that contained questions related to effort. We collected data on student effort by asking students the following radio button style question upon the completion of the TOSLS assessment: On a scale of 0–10 indicate the level of effort you exerted on this assessment (0 = none, 10 = maximum effort). Self-reported effort values are an effective way to measure effort, yielding similar values to other methods such as response time effort (RTE) [11].

Prior to the initiation of the test, students in graded sections were informed that the percentage of correct answers from the TOSLS assessment would count as a quiz grade (approximately 0.75–3% of the overall course grade). Because the students would not be given the opportunity to prepare for the TOSLS, we expected to encounter some testing anxiety amongst the grade-conscious members of the class. In an effort to minimize the detrimental effects of testing anxiety, we announced our grading plan to scale the mean score of each section to 90%, with a maximum score of 100%. In both graded and ungraded sections, students were verbally encouraged to take the test seriously by emphasizing the importance of the TOSLS assessment to departmental and university-level curricular decisions.

#### Data analysis

These experiments and their analysis were not blinded/randomized. Data were tested for normality using Shapiro–Wilk test using JMP (Statistical Analysis Software, Cary, NC), and normality was determined as P > 0.05. When data were not normally distributed and could not be transformed to achieve normality, non-parametric analyses were used.

Mean test scores were compared using a mixed repeated-measures ANOVA in SPSS (SPSS Inc., Chicago, IL, USA). Grade condition (grade/no grade) was used as the between-subjects factor, and test condition (pretest/posttest) as the within-subjects factor. The interaction between grade and test condition was also tested in the same model. One-tailed, paired t-tests were used as *post-hoc* tests to determine which group(s) showed significant gains between the pretest and posttest. Mean effort on the pretest and posttest for the grade/no grade groups was compared using Kruskal–Wallis test in StatsDirect (StatsDirect, Cheshire, UK).

A non-parametric linear regression was used to examine the correlation between effort and test score for each of the four groups (grade/no grade and pretest/posttest) using StatsDirect software. In all analyses, P values less than 0.05 were considered significant.

### Results

Pre and post-scores for both groups were normally distributed. There was no significant effect of grade condition (grade/no grade) on test score (F(1,162) = 0.665, P = 0.416). There was a significant effect of test condition (pretest/posttest) on test scores (F(1,162) = 12.2, P = 0.001), with posttests being significantly higher than pretests. There was no significant interaction effect of grade condition (grade/no grade) and test condition (pretest/posttest) on test score (F(1,162) = 0.004, P = 0.949). Paired t-tests showed that both grade and no grade groups showed significant gains between the pretest and posttest (P < 0.01 for no grade, P = 0.005 for grade).

**Fig. 1:**
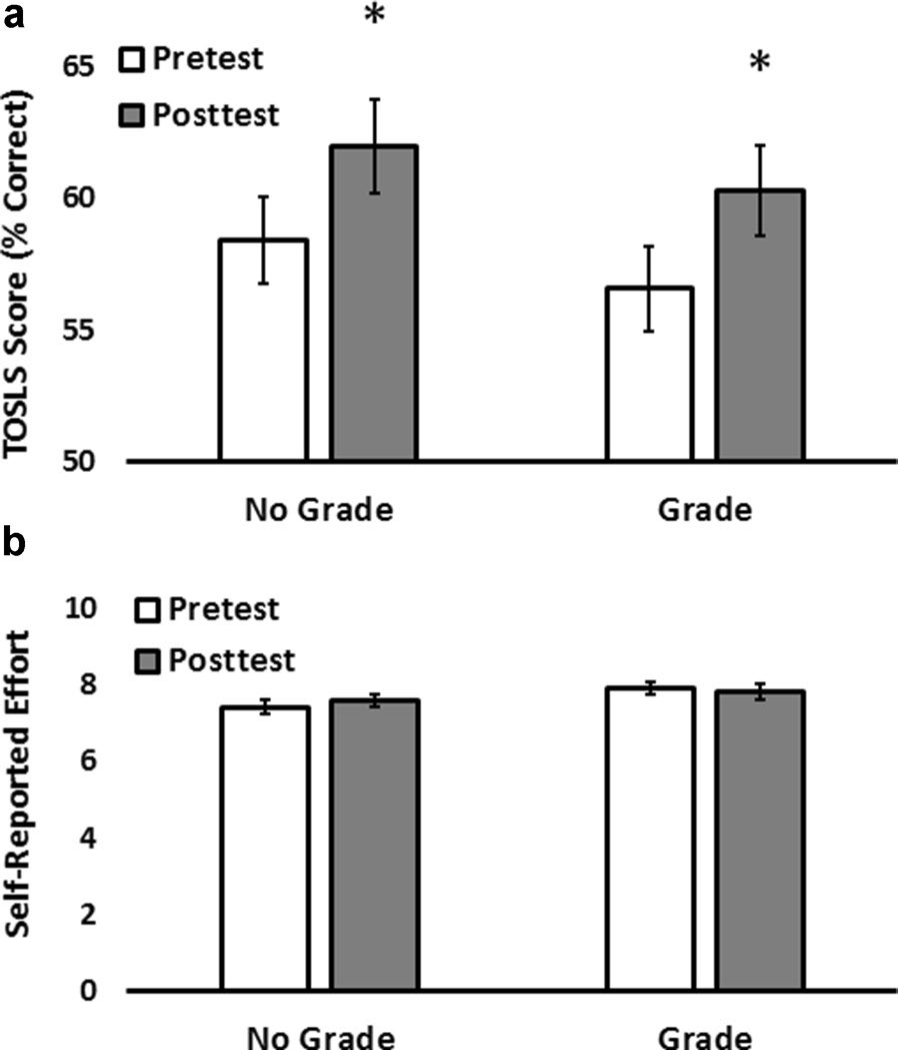
Assigning a grade had no significant effect on student performance (**a**) or self-reported effort (**b**) on the TOSLS within our general education Biology course. Repeated measures ANOVA showed no significant effect of grade/no grade on score (P = 0.416) or self-reported effort (P = 0.18). There was a significant difference between the pretest and posttest scores in both models (P = 0.001). Pair-wise comparisons were made with post-hoc, paired t-tests. Error bars represent standard error of the mean. Asterisks denote significant differences between pretest and posttest scores as determined by *post hoc* paired t-tests. P < 0.01 for no grade, and 0.005 for grade

There was no significant difference in effort reported by students in any of the four test groups (Fig. 1b; P = 0.18).

### Discussion

We use the TOSLS assessment every semester to measure student gains in scientific literacy in the context of our general education Biology courses. In general, this is done by administering a pre-semester and post-semester TOSLS assessment and looking for a statistically significant increase in mean student scores. We use this data to inform curricular changes and innovations that favor gains in scientific literacy.

**Fig. 2:**
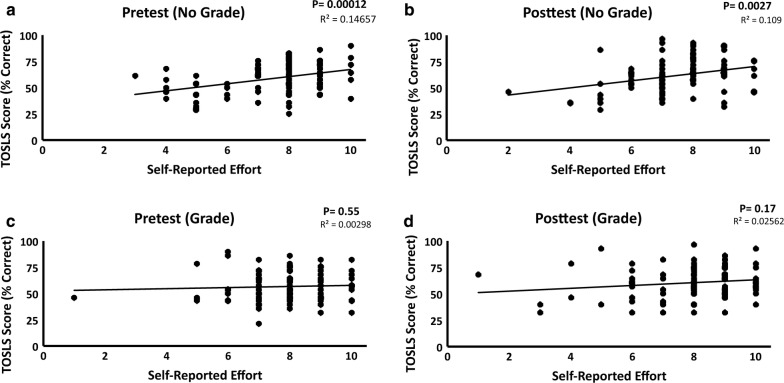
Relationship between TOSLS score and self-reported effort. We detected a positive correlation between self-reported effort and test score in both the pretest and the posttest groups of the ungraded cohort (**a**, **b**). No correlation was observed in the graded cohort (**c**, **d**). The relationship between score and self-reported effort was analyzed using a linear regression model. We judged statistical significance to be P < 0.05

We saw no difference in the average student TOSLS scores (Fig. 1a) or self-reported student effort (Fig. 1b) when comparing the sections that received a grade and those that did not. These data indicate that assigning a grade had no significant effect on mean student performance or student self-reported effort on the TOSLS within our general education course in the context of the described TOSLS grading policy. Interestingly, we found that there was a significant, positive correlation between self-reported effort and test score in both the pretest and posttest groups in the ungraded cohort (Fig. 2a, b). However, this relationship was absent in the graded sections (Fig. 2c, d). While our study did not include an assessment of testing anxiety, it is a well-documented phenomenon that has been reported to reduce testing performance [12]. Thus, stress derived from our grading policy and resulting reduction in testing performance could account for the lack of association between self-reported effort and TOSLS performance in the graded cohort.

Alternative TOSLS grading policies that could be tested in the future include not curving the quiz at all, increasing the portion of the course grade that relies on TOSLS performance, and having the TOSLS performance grade be based off of improvement on the TOSLS raw score when comparing pre/post-examinations. Would changes in our grading policy affect TOSLS scores or self-reported student effort? While it is impossible to predict changes in TOSLS student scores as a response to further increases in stakes or value, previous literature in the field shows that, in the context of low-stakes testing, the cognitive demand of a particular assessment trumps the value students assign to a particular assessment [10].

Overall, these results suggest that in spite of a visible lack of student investment in ungraded assessments, the process of standardized testing before and after course completion still provides a relatively consistent and accurate measure of student gains.

## Limitations

This study was conducted at a single university, and results may not be typical of other universities with different student populations.

## Abbreviations

TOSLS: Test of Scientific Literacy Skills
V&C: Vision & Change in Undergraduate Biology Education Initiative
